# Expression and clinical value of CXCR4 in high grade gastroenteropancreatic neuroendocrine neoplasms

**DOI:** 10.3389/fendo.2024.1281622

**Published:** 2024-03-08

**Authors:** Chaoyu Pang, Yongzheng Li, Ming Shi, Zhiyao Fan, Xin Gao, Yufan Meng, Shujie Liu, Changhao Gao, Peng Su, Xiao Wang, Hanxiang Zhan

**Affiliations:** ^1^ Division of Pancreatic Surgery, Department of General Surgery, Qilu Hospital of Shandong University, Jinan, Shandong, China; ^2^ Department of Pathology, Qilu Hospital of Shandong University, Jinan, Shandong, China

**Keywords:** GEP-NEN G3, neuroendocrine neoplasm, CXCR4, clinicopathological features, prognosis

## Abstract

**Background:**

CXC chemokine receptor 4 (CXCR4) is associated with the progression and metastasis of numerous malignant tumors. However, its relationship with Gastroenteropancreatic Neuroendocrine Neoplasms Grade 3 (GEP-NENs G3) is unclear. The aim of this study was to characterize the expression of CXCR4 in GEP-NENS and to explore the clinical and prognostic value of CXCR4.

**Methods:**

This study retrospectively collected clinical and pathological data from patients with GEP-NENs who receiving surgery in Qilu Hospital of Shandong University from January 2013 to April 2021, and obtained the overall survival of the patients based on follow-up. Immunohistochemistry (IHC) was performed on pathological paraffin sections to observe CXCR4 staining. Groups were made according to pathological findings. Kaplan-Meier (K-M) curve was used to evaluate prognosis. SPSS 26.0 was used for statistical analysis.

**Results:**

100 GEP-NENs G3 patients were enrolled in this study. There was a significant difference in primary sites (P=0.002), Ki-67 index (P<0.001), and Carcinoembryonic Antigen (CEA) elevation (P=0.008) between neuroendocrine tumor (NET) G3 and neuroendocrine carcinoma (NEC). CXCR4 was highly expressed only in tumors, low or no expressed in adjacent tissues (P<0.001). The expression level of CXCR4 in NEC was significantly higher than that in NET G3 (P=0.038). The K-M curves showed that there was no significant difference in overall survival between patients with high CXCR4 expression and patients with low CXCR4 expression, either in GEP-NEN G3 or NEC (P=0.920, P=0.842. respectively).

**Conclusion:**

Differential expression of CXCR4 was found between tumor and adjacent tissues and between NET G3 and NEC. Our results demonstrated that CXCR4 can be served as a new IHC diagnostic indicator in the diagnosis and differential diagnosis of GEP-NENs G3. Further studies with multi-center, large sample size and longer follow-up are needed to confirm the correlation between CXCR4 expression level and prognosis.

## 1 Introduction

Neuroendocrine neoplasms (NENs) are highly heterogeneous solid tumors originating from the diffuse neuroendocrine system. They have neuroendocrine markers and can produce hormones such as peptides and biogenic amines (1, 2). Gastroenteropancreatic neuroendocrine neoplasms (GEP-NENs) accounts for about 60% of all NENs (3). The incidence of NENs has increased remarkably in recent years. Statistics from the Surveillance, Epidemiology, and End Results Database (SEER) showed that the incidence of GEP-NENs increased from 1.09/100,000 in 1973 to 6.98/100,000 in 2012 (4).

The classification of NENs is based on tumor proliferation activity (including Ki-67 index and mitotic rate), pathological morphology, and immunohistochemical (IHC) staining. 2019 WHO Classification of Digestive System Tumors (5th Edition) updated and defined a new subtype, GEP-NET G3. NENs can be classified into G1, G2 and G3 based on tumor proliferation activity, and G3 includes well-differentiated NET G3 and poorly-differentiated NEC (5). Few studies have been carried out focusing on the similarities and differences between well-differentiated NET G3 and poorly-differentiated NEC up to now. There are significant differences between NET G3 and NEC in terms of treatment. For instance, the European Society for Medical Oncology considers locally advanced NEC or NEC with distant metastasis as a contraindication for surgery, but NET G3 can be considered for surgical treatment (6). Therefore, accurate identification of GEP-NET G3 and NEC is of great significance for the selection of following treatment. However, the accuracy of existing immunohistochemical diagnostic indicators such as Rb, P53, DAXX/ATRX, and SSTR2 in the identification of NET G3 and NEC is not satisfactory (7–9).

Chemokines are a kind of small protein peptides that have directional chemotactic effects on neutrophils and monocytes. CXC chemokine receptor 4 (CXCR4), which belongs to the CXCR subfamily and is a G protein-coupled sevenfold transmembrane receptor. The coding gene is located on chromosome 2q21. Its N-terminal is located extracellular and is the binding site of the only ligand CXCL12 (CXC chemokine Ligand 12), while its C-terminal is located intracellular and contains Ser/Thr site, which is involved in intracellular signal transduction (10, 11). CXCR4 is associated with the occurrence, progression, invasion, and metastasis of a variety of malignant tumors according to previous literatures (12–16). CXCR4 has also been considered a prognostic factor in NENs, its expression level correlates with the malignancy of NENs (17, 18). In a retrospective study, Popa et al. found that high CXCR4 expression was associated with high-grade advanced NENs and tumor metastasis (18).

This study aimed to detect the expression pattern of CXCR4 in patients with NET G3 and NEC, to correlate the level of expression with clinicopathological characteristics and survival, and to provide preliminary validation results for the diagnosis, differential diagnosis, prognosis prediction and targeted therapy of GEP-NEN G3.

## 2 Methods

### 2.1 Study design

This study was a single-center, retrospective, observational study involving patients with GEP-NENs G3 who were admitted to Qilu Hospital of Shandong University from January 2013 to April 2021. We comprehensively collected patient information, including (1) Clinical data: age, gender, tumor markers. (2) Pathological data: tumor primary site, tumor size, stage. (3) Prognostic data: Overall survival (OS) time was defined as the time from surgery to death from any cause. At the same time, IHC staining of tumor marker CXCR4 was performed on the pathological specimens of all patients for further statistical analysis. The 2019 WHO Classification of Digestive System (5th Edition) was used for diagnosis and staging of NENs (5). By retrospectively collecting pathological data, GEP-NENs were classified into NET G3 and NEC by two experienced pathologists. This research has passed the review of the Ethics Committee of Qilu Hospital of Shandong University. The Study was Conducted in accordance with the Declaration of Helsinki (revised in 2013).

### 2.2 Case selection

**Figure 1:**
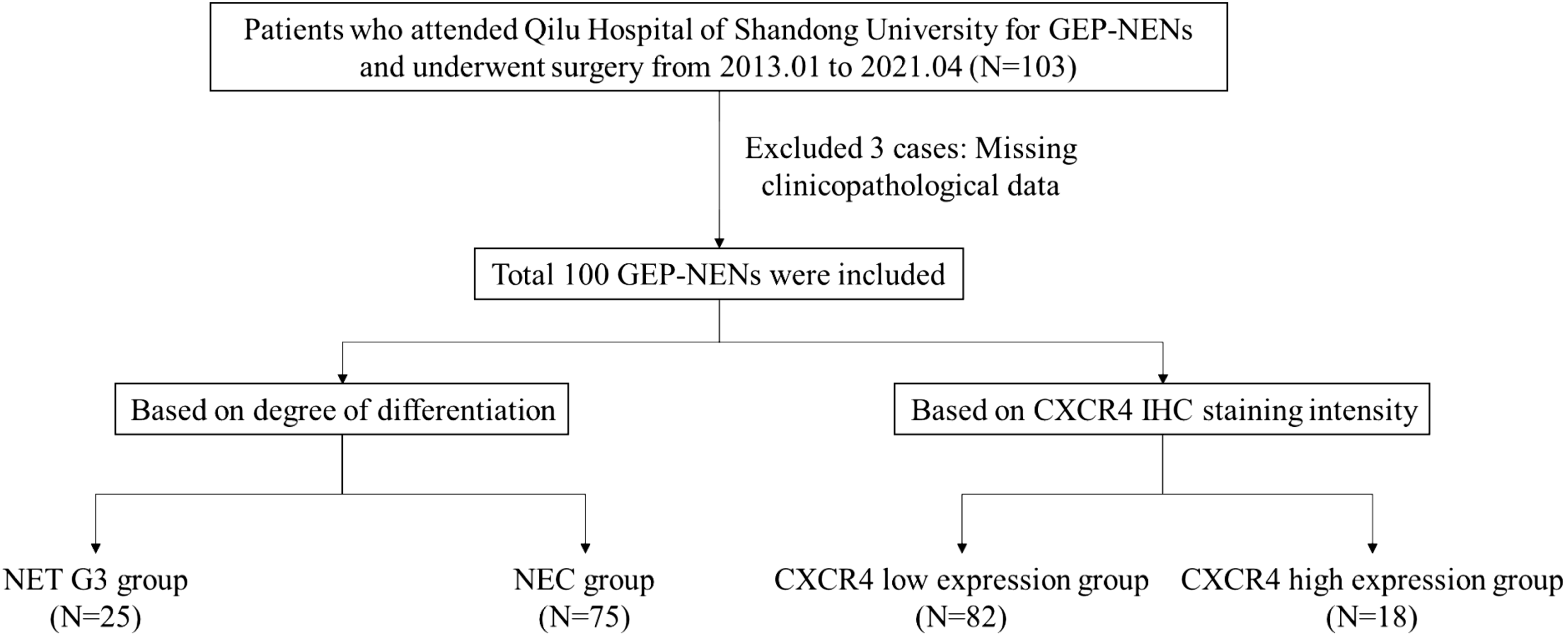
Patient cohort selection flow. A total of 100 participants were enrolled in this study.

This study followed the following inclusion criteria: (1) Patients who received surgical treatment in Qilu Hospital of Shandong University. (2) Pathology confirmed that the lesion is GEP-NENs G3. (3) Patients who never received neoadjuvant therapy before surgical treatment. (4) Patients who have complete clinicopathological data. Exclusion criteria: (1) Age < 18 years old. (2) Combined with other malignant tumors. (3) Multiple neuroendocrine neoplasia, such as multiple endocrine neoplasia type 1 (MEN1), multiple endocrine neoplasia type 2 (MEN2), etc. A total of 100 participants were enrolled in this study, the flowchart of the study was shown in **Figure 1**.

### 2.3 Sample collection

Collecting serum collection before receiving treatment, the serum tumor markers, including carbohydrate antigen (CA)19-9, CA 125, carcinoembryonic antigen (CEA), alpha fetoprotein (AFP) and so on were measured after at least 12 hours of fasting. Due to the heterogeneity of NENs, we selected surgically resected specimens instead of biopsy specimens for IHC staining to ensure a more comprehensive reflection of CXCR4 expression in tumor tissues and adjacent tissues. For cases with metastatic disease, we selected only the primary tumor site because there is insufficient evidence to show the consistency of CXCR4 expression between the primary tumor and metastatic lesions.

### 2.4 Immunohistochemistry

The formalin-fixed paraffin-embedded tumor tissues were obtained from Department of Pathology, Qilu Hospital of Shandong University. The wax block tissue was cut into continuous tissue sections with a thickness of 4 microns by microtome, and then roasted in a drying instrument at 65°C for 2 hours until dewaxing. The sections were successively dewaxed with xylene, 100% alcohol, 90% alcohol, 80% alcohol, 70% alcohol, and finally washed three times with phosphate buffer solution (PBS). Antigen repair was performed using EDTA solution. An appropriate amount of endogenous peroxidase blocker was added to the tissue and incubated for 15 minutes at room temperature. Primary antibody Anti-CXCR4 (1:200, ab181020; EPUMBR3) was added and incubated overnight. After removing the antibody solution, use PBS to clear it three times, each time lasting 3 minutes. Drop an appropriate amount of reaction booster solution, incubate at room temperature for 20 minutes, and then wash with PBS for 3 times. After that, secondary antibody was added, and the samples were incubated at room temperature for 20 minutes, and washed with PBS for 3 times. Positive control (colon cancer tissue section) and negative control (PBS instead of primary antibody) were set for each batch.

### 2.5 Scoring methods

All immuno-stained sections and matched hematoxylin and eosin stained sections were scanned by optical microscopy and were evaluated by the intensity (negative scored as 0, weak scored as 1, moderate scored as 2, strong scored as 3) by two experienced pathologists. Score 0-1 was divided into CXCR4 low expression group, and score 2-3 was divided into CXCR4 high expression group.

### 2.6 Statistical analysis

SPSS 26.0 and GraphPad Prism 8.0 were used for data sorting and analyzing. Data were expressed as the number of cases and the rate. Continuous variables were described in terms of median and interquartile range (IQR) and were compared using the Mann-Whitney U test. Categorical variables were compared using the χ2 test. Overall survival of GEP-NEN G3 patients was estimated by using the K-M method and log-rank test. P<0.05 was considered statistically significant.

## 3 Results

### 3.1 Clinicopathological features and prognosis of GEP-NEN G3 patients

A total of 100 GEP-NENs G3 patients (25 NETs G3 and 75 NECs) were ultimately enrolled in this study. The comparison of clinicopathological characteristics between NET G3 and NEC are summarized in **Table 1**. The mean age of all patients was 63.1 years old, there was no significant difference in age between NET G3 and NEC (P=0.109) and in gender composition (P=0.330). The primary sites of NET G3 were stomach (11/25, 44.0%), pancreas (9/25, 36.0%), esophagus (2/25, 8.0%), gallbladder (2/25, 8.0%), and colorectal (1/25, 4.0%), in order. And the primary sites of NEC were stomach (40/75, 54.1%), esophagus (12/75, 16.2%), duodenum and small intestine (8/75, 10.8%), colorectal (7/75, 9.5%), gallbladder (4/75, 5.4%) and pancreas (3/75, 4.1%), in order. The primary site was significantly different between NET G3 and NEC (P=0.002). No matter in NEC or in NET G3, the most common primary sites were both stomachs. The maximum diameter of the tumor specimens ranged from 0.9 to 17.0 centimeters (cm), with a mean of 4.77 cm. There was no significant difference between the two groups in terms of maximum diameter of the tumor (P=0.149). The mean Ki-67 value for all specimens was 57.6%, including 39.2% for NET G3 and 65.1% for NEC. We found that among NET G3 patients, 4 cases (16.7%) have Ki-67 ≥ 55%, which significantly lower than that of patients with NEC (74.7%, 56/75) (P < 0.001). There were no significant differences between NET G3 and NEC patients in tumor function, lymph node metastasis, liver metastasis, vascular invasion, perineural infiltration, clinical stage (**Table 1**).

**Table 1:** Comparison of clinicopathological parameters between NET G3 and NEC.

Variables	n	NET G3 (n=25)	NEC (n=75)	P value
Age (years)		59.0 (49.0-68.5)	63.0 (59.0-67.0)	0.109
Tumor size (cm)		4.00 (2.50-5.00)	4.70 (2.70-6.50)	0.149
Gender, n (%)				0.330
Male	86	20(80.0%)	66(88.0%)	
Female	14	5(20.0%)	9(12.0%)	
Tumor site ^a^, n (%)				0.002
Esophagus	14	2(8.0%)	12(16.2%)	
Stomach	51	11(44.0%)	40(54.1%)	
Pancreas	12	9(36.0%)	3(4.1%)	
Colorectum	8	1(4.0%)	7(9.5%)	
cholecyst	6	2(8.0%)	4(5.4%)	
Duodenum & small intestine	8	0(0.0%)	8(10.8%)	
Functional or not, n (%)				0.250
No	99	24(96.0%)	75(100.0%)	
Yes	1	1(4.0%)	0(0.0%)	
Tumor size ^b^, n (%)				0.051
<5 cm	53	17(73.9%)	36(50.7%)	
≥5 cm	41	6(26.1%)	35(49.3%)	
Ki-67% ^c^, n (%)				0.000
<55%	39	20(83.3%)	19(25.3%)	
≥55%	60	4(16.7%)	56(74.7%)	
Lymph node metastasis ^d^, n (%)				0.078
No	26	9(45.0%)	17(24.6%)	
Yes	63	11(55.0%)	52(75.4%)	
Liver metastasis, n (%)				0.115
No	90	20(80.0%)	70(93.3%)	
Yes	10	5(20.0%)	5(6.7%)	
Perineural infiltration ^e^, n (%)				0.262
No	9	1(25.0%)	8(66.7%)	
Yes	7	3(75.0%)	4(33.3%)	
Vascular invasion ^f^, n (%)				0.387
No	8	1(9.1%)	7(29.2%)	
Yes	27	10(90.9%)	17(70.8%)	
Clinical stage ^g^, n (%)				0.499
I + II	32	9(39.1%)	23(31.5%)	
III + IV	64	14(60.9%)	50(68.5%)	

^a^ Tumor site data were missing in 1 case. ^b^ Tumor size data were missing in 6 cases. ^c^ Ki-67 data were missing in 1 case. ^d^ Lymph node metastasis data were missing in 11 cases. ^e^ Perineural infiltration data were missing in 84 cases. ^f^ Vascular invasion data were missing in 65 cases. ^g^ Clinical stage data were missing in 4 cases.

For serum tumor markers, all NET patients (17/17, 100%) have normal CEA, while only 69.8% of NEC patients (37/53) have normal CEA, and the difference between them was statistically significant (P=0.008) (**Table 2**).We also find that the expression level of CA125 was increased in 4 patients and the expression level of AFP was increased in 3 patients, all of whom were NEC patients, but the results were not statistically different. Furthermore, there were no significant differences in other serum tumor markers between NET G3 patients and NEC patients.

**Table 2:** Comparison of serum tumor markers between NET G3 and NEC (n=72).

Variables	n	NET G3	NEC	P value
CA19-9 (U/ml) ^a^				0.676
≤37	63	16(25.4%)	47(74.6%)	
>37	9	1(11.1%)	8(88.9%)	
CA125 (U/ml) ^b^				0.565
≤35	63	17(27.0%)	46(73.0%)	
>35	4	0(0.0%)	4(100%)	
CEA (ng/ml) ^c^				0.008
≤5	54	17(31.5%)	37(68.5%)	
>5	16	0(0.0%)	16(100%)	
CA724(U/ml) ^d^				0.367
≤6.9	51	8(15.7%)	43(84.3%)	
>6.9	10	3(30.0%)	7(70.0%)	
AFP (ng/ml) ^e^				0.569
≤20	62	16(25.8%)	46(74.2%)	
>20	3	0(0.0%)	3(100%)	
NSE (ng/ml) ^f^				0.676
≤20	23	9(39.1%)	14(60.9%)	
>20	8	2(25.0%)	6(75.0%)	
SA (mg/dl) ^g^				0.663
≤75.4	40	9(22.5%)	31(77.5%)	
>75.4	10	1(10.0%)	9(90.0%)	
SCC (ng/ml) ^h^				1.000
≤1.5	56	15(26.8%)	41(73.2%)	
>1.5	0	0(0.0%)	0(0.0%)	

CA19-9, carbohydrate antigen 19-9; CA125, carbohydrate antigen 125; CEA, carcinoembryonic antigen; CA724, carbohydrate antigen 724; AFP, alpha fetoprotein; NSE, neuron specific enolase; SA, sialic acid; SCC, squamous cell carcinoma. ^a^ CA19-9 data were missing in 28 cases. ^b^ CA125 data were missing in 33 cases. ^c^ CEA data were missing in 30 cases. ^d^ CA724 data were missing in 39 cases. ^e^ AFP data were missing in 35 cases. ^f^ NSE data were missing in 69 cases. ^g^ SA data were missing in 50 cases. ^h^ SCC data were missing in 44 cases.

**Figure 2:**
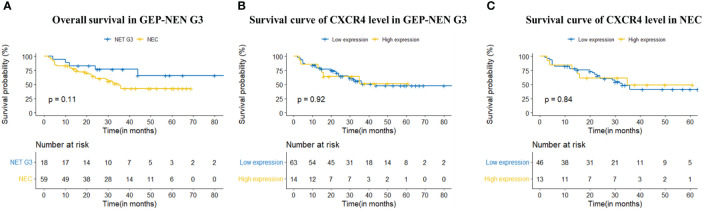
K-M Survival curve. **(A)** Kaplan-Meier survival curve showed that the prognosis of NET G3 was better than NEC but there is no statistical difference between the two group (P = 0.110); **(B, C)** The K-M curves showed that there was no significant difference in overall survival between patients with high CXCR4 expression and patients with low CXCR4 expression, either in GEP-NEN G3 or NEC (P=0.920, P=0.842. respectively).

In terms of prognosis, A total of 34 patients died during the follow-up period, including 5 (14.7%) cases with NET G3 and 29 (85.3%) cases with NEC. The median OS for patients with NEC was 35.0 m (95%CI, 28.7m-41.3m), while NET G3 patients did not reach the median follow-up time by the end of the study. Log-rank test showed there was no statistical difference between the two group (P = 0.110) (**Figure 2A**).

### 3.2 The expression of CXCR4 in NET G3 and NEC

**Figure 3:**
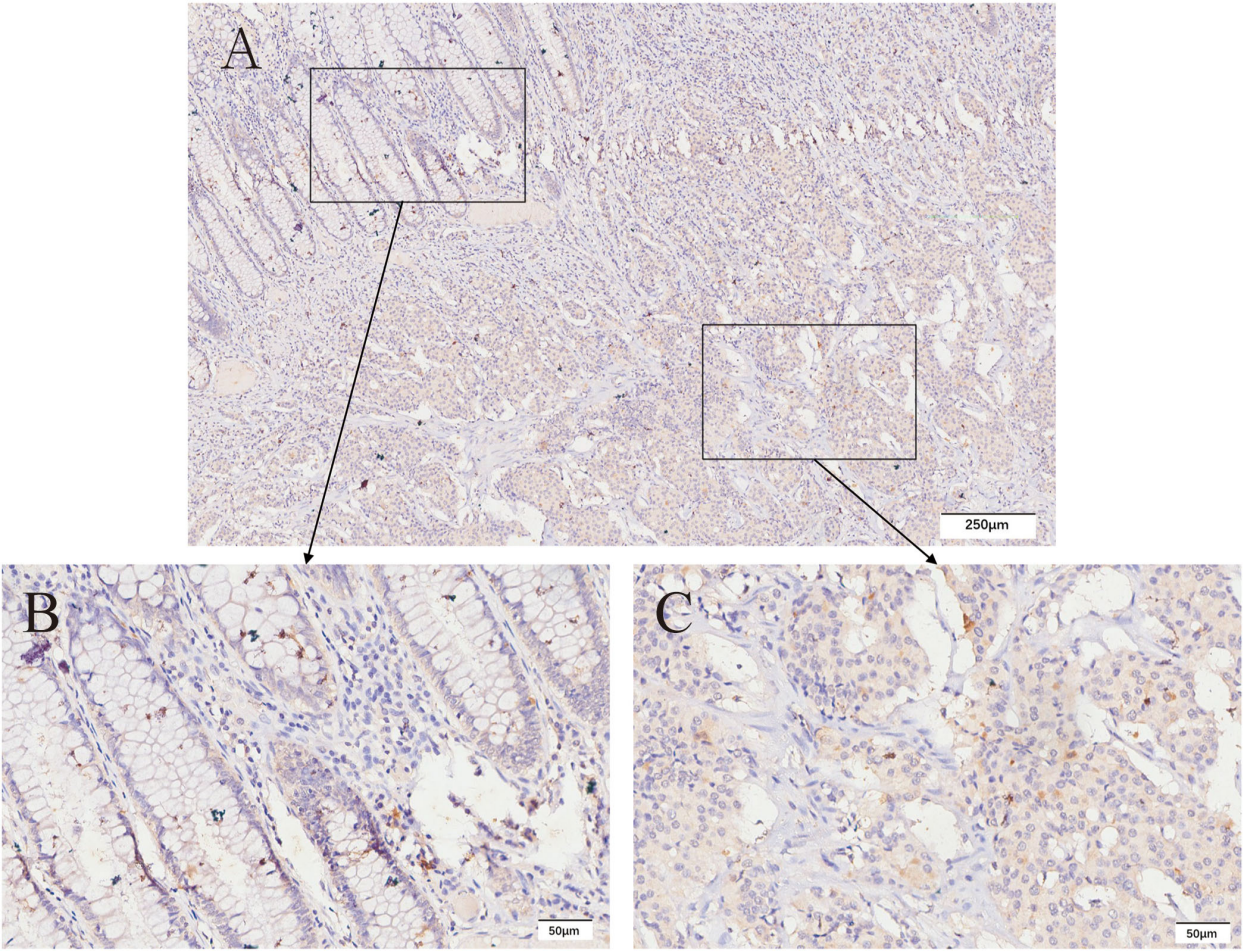
Expression of CXCR4 in rectal NET G3. Immunohistochemical detection of CXCR4 expression in rectal NET G3, CXCR4 is mainly expressed in the membrane and cytoplasm of tumor cells. CXCR4 was lowly expressed in adjacent tissues **(B)** compared with tumor tissues **(C)**. Original magnifications×40 **(A)**, ×200 **(B, C)**.

**Figure 4:**
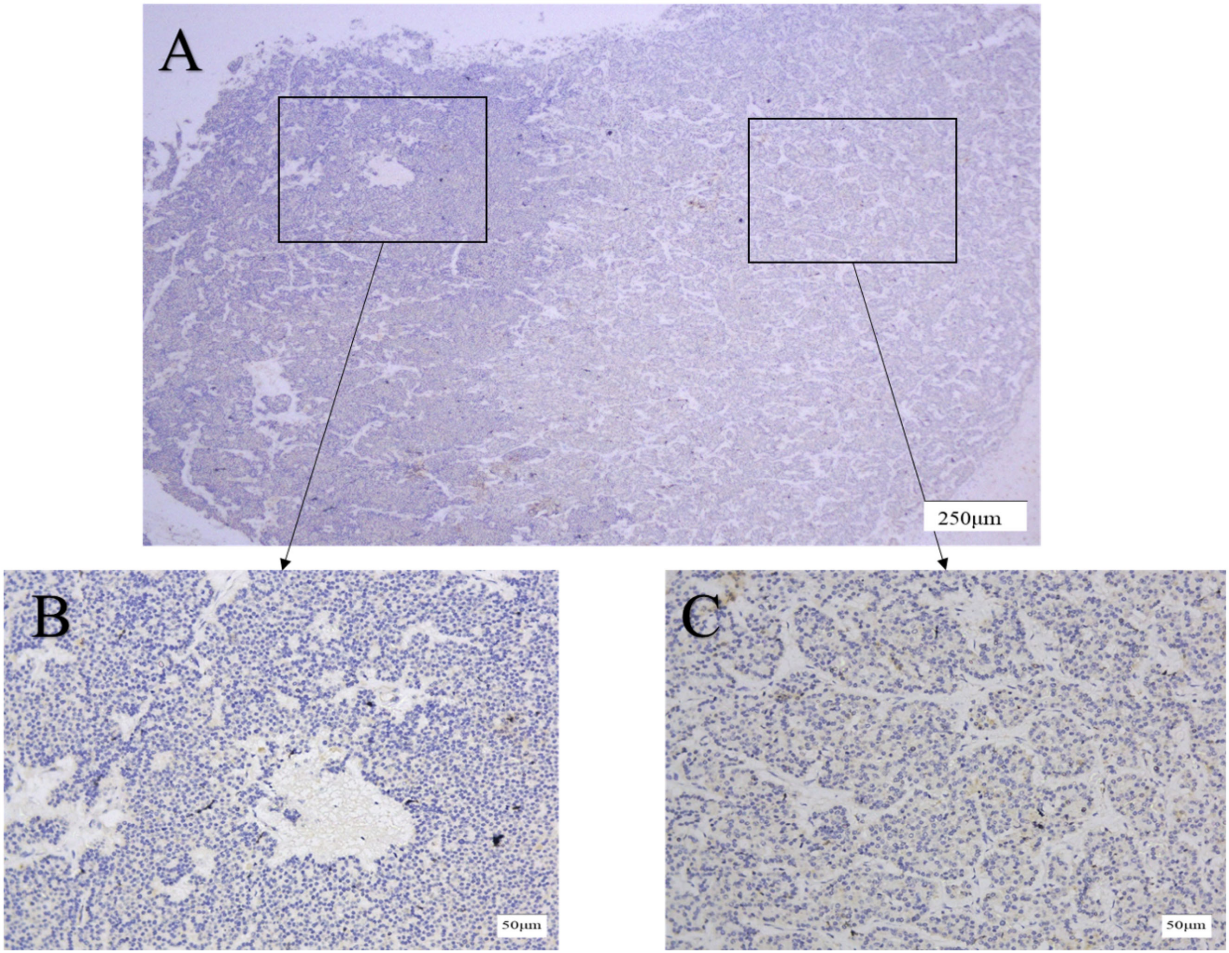
Expression of CXCR4 in pancreatic NET G3. Immunohistochemical detection of CXCR4 expression in pancreatic NET G3, CXCR4 is mainly expressed in the membrane and cytoplasm of tumor cells. CXCR4 was lowly expressed in adjacent tissues **(B)** compared with tumor tissues **(C)**. Original magnifications×40 **(A)**, ×200 **(B, C)**.

**Figure 5:**
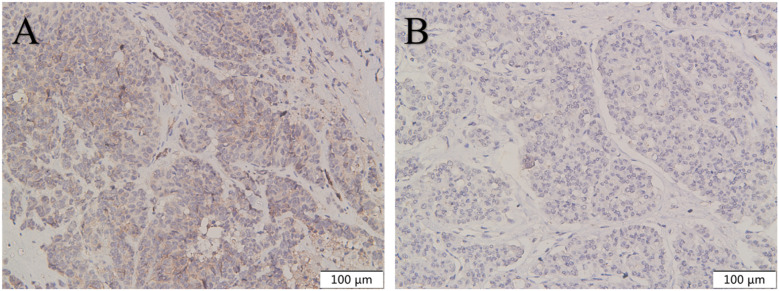
The expression of CXCR4 in pancreatic NEC was higher than in pancreatic NET G3. CXCR4 was highly expressed in pancreatic NEC **(A)** compared with pancreatic NET G3 **(B)**. Original magnifications×100.

**Figure 6:**
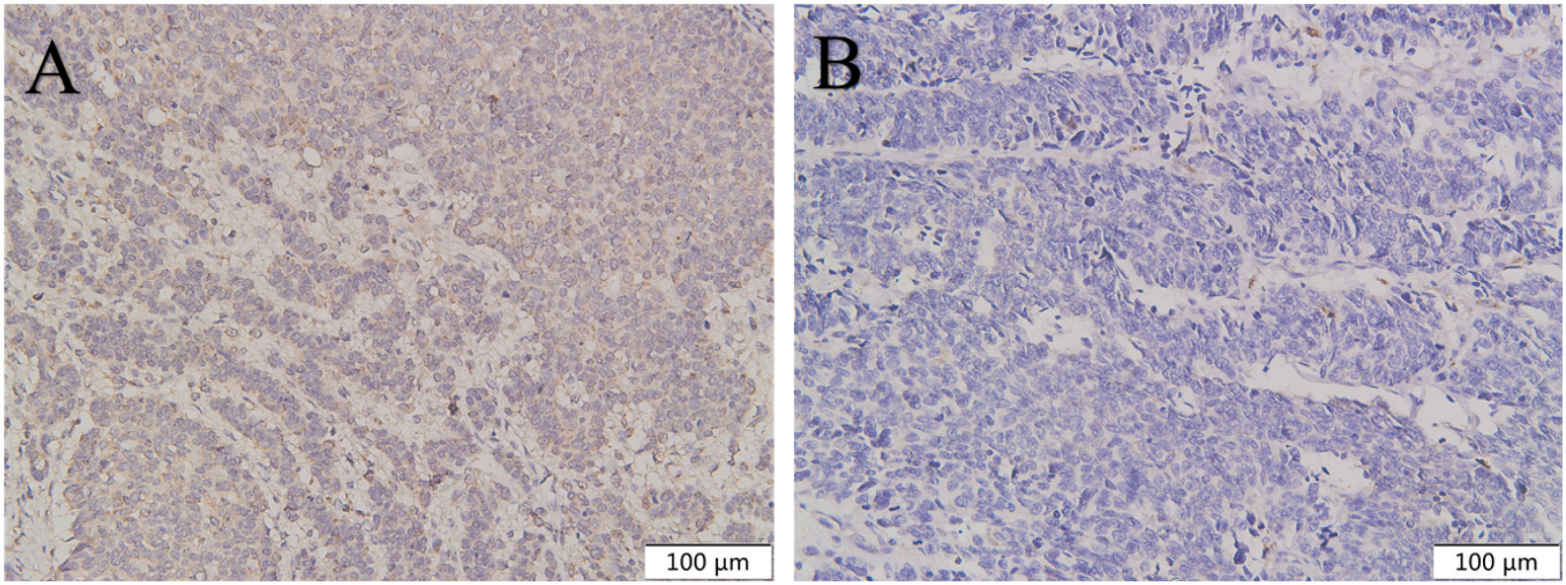
The expression of CXCR4 in gastric NEC was higher than in gastric NET G3. CXCR4 was highly expressed in gastric NEC **(A)** compared with gastric NET G3 **(B)**. Original magnifications×100.

CXCR4 is mainly expressed in the membrane and cytoplasm of tumor cells, and cytoplasmic coloration reflects the internalization of the receptor in response to agonist stimulation (19). Both membrane and cytoplasmic coloration were evaluated equally in this study. Among GEP-NEN G3 patients, 82 cases were classified into CXCR4 low expression group (82.0%), and 18 cases were classified into CXCR4 high expression group (18.0%). The expression of CXCR4 in the adjacent tissues was low or none, and the expression of CXCR4 was significantly different between cancer and adjacent tissues (P < 0.001). In the CXCR4 low expression group, NET G3 accounted for 29.3% (24/82) and NEC accounted for 70.7% (58/82). In the CXCR4 high expression group, 17 cases (94.4%) were NEC and only 1 case (5.6%) was NET G3. The high expression rate of CXCR4 in NEC was 5.7 times higher than that in NET G3 (17/75 *vs* 1/25, P=0.038). **Figures 3**–**6** shows the expression of CXCR4 in GEP-NEN G3 of different tumor origins and in tumor and adjacent tissues.

### 3.3 Relationship between the expression level of CXCR4 and clinicopathological features in NEC and GEP-NEN G3

The expression level of CXCR4 in NEC patients with different tumor origins was significantly different (P=0.013). The proportion of CXCR4 overexpression was the highest in pancreatic NEC patients (66.7%), followed by colorectal (57.1%), gallbladder (50.0%), esophagus (25.0%) and stomach (15.0%). The expression level of CXCR4 in NEC patients was not significantly correlated with gender, tumor size and other clinicopathological parameters (**Table 3**).

**Table 3:** Relationship between the expression level of CXCR4 and clinicopathological features in NEC patients.

Variables	n	CXCR4 low expression group (n=58)	CXCR4 high expression group (n=17)	P value
Gender, n (%)				1.000
Male	66	51(77.3%)	15(22.7%)	
Female	9	7(77.8%)	2(22.2%)	
Tumor site ^a^, n (%)				0.013
Esophagus	12	9(75.0%)	3(25.0%)	
Stomach	40	34(85.0%)	6(15.0%)	
Pancreas	3	1(33.3%)	2(66.7%)	
Colorectum	7	3(42.9%)	4(57.1%)	
cholecyst	4	2(50.0%)	2(50.0%)	
Duodenum & small intestine	8	8(100.0%)	0(0%)	
Tumor size ^b^, n (%)				0.725
<5 cm	36	29(80.6%)	7(19.4%)	
≥5 cm	35	27(77.1%)	8(22.9%)	
Ki-67%, n (%)				1.000
<55%	19	15(78.9%)	4(21.1%)	
≥55%	56	43(76.8%)	13(23.2%)	
Lymph node metastasis ^c^, n (%)				0.734
No	17	13(76.5%)	4(23.5%)	
Yes	52	42(80.8%)	10(19.2%)	
Liver metastasis, n (%)				1.000
No	70	54(77.1%)	16(22.9%)	
Yes	5	4(80.0%)	1(20.0%)	
Clinical stage ^d^, n (%)				0.233
I + II	23	16(69.6%)	7(30.4%)	
III + IV	50	41(82.0%)	9(18.0%)	

^a^ Tumor site data were missing in 1 case. ^b^ Tumor size data were missing in 4 cases. ^c^ Lymph node metastasis data were missing in 6 cases. ^d^ Clinical stage data were missing in 2 cases.

We further explore the relationship between CXCR4 expression and clinicopathological features in patients with GEP-NEN G3 (**Table 4**). However, there is no statistically significant difference in the expression level of CXCR4 between tumors of different primary sites (P = 0.057). We find that the high expression rate of CXCR4 in patients with Ki67 index ≥ 55% was higher than that in patients with Ki67 index < 55%, although the difference was not statistically significant (21.6% *vs* 12.8%, P = 0.265). The expression level of CXCR4 was not significantly correlated with gender, lymph node metastasis, liver metastasis and clinical stage (**Table 4**). The K-M curves showed that there was no significant difference in OS between patients with high CXCR4 expression and patients with low CXCR4 expression, either in GEP-NEN G3 or NEC (P=0.920, P=0. 842. respectively) (**Figures 2B, C**).

**Table 4:** Relationship between the expression level CXCR4 and clinicopathological features in GEP-NEN G3 patients.

Variables	n	CXCR4 low expression group (n=82)	CXCR4 high expression group (n=18)	P value
Gender, n (%)				1.000
Male	86	70(81.4%)	16(18.6%)	
Female	14	12(85.7%)	2(14.3%)	
Tumor site ^a^, n (%)				0.057
Esophagus	14	11(78.6%)	3(21.4%)	
Stomach	51	45(88.2%)	6(11.8%)	
Pancreas	12	9(75.0%)	3(25.0%)	
Colorectum	8	4(50.0%)	4(50.0%)	
cholecyst	6	4(66.7%)	2(33.3%)	
Duodenum & small intestine	8	8(100%)	0(0.0%)	
Tumor size ^b^, n (%)				0.572
<5 cm	53	45(84.9%)	8(15.1%)	
≥5 cm	41	33(80.5%)	8(19.5%)	
Ki-67% ^c^, n (%)				0.265
<55%	39	34(87.2%)	5(12.8%)	
≥55%	60	47(78.3%)	13(21.6%)	
Lymph node metastasis ^d^, n (%)				1.000
No	26	22(84.6%)	4(15.4%)	
Yes	63	52(70.3%)	11(73.3%)	
Liver metastasis, n (%)				1.000
No	90	74(82.2%)	16(17.8%)	
Yes	10	8(80.0%)	2(20.0%)	
Clinical stage ^e^, n (%)				
I + II	32	25(78.1%)	7(21.9%)	0.450
III + IV	64	54(84.4%)	10(15.6%)	

^a^ Tumor site data were missing in 1 case. ^b^ Tumor size data were missing in 6 cases. ^c^ Ki-67 data were missing in 1 case. ^d^ Lymph node metastasis data were missing in 11 cases. ^e^ Clinical stage data were missing in 4 cases.

## 4 Discussion

The origin, molecular mechanism, biological behavior, histopathological features, clinical manifestations, treatment and prognosis of NET G3 are different from those of NEC (2, 20). This study systematically analyzed the clinicopathological features and prognosis and investigated the expression of CXCR4 in tumor and adjacent tissues of patients with GEP-NENs G3 who underwent surgical treatment in Qilu Hospital of Shandong University. We hope our study can provide reference for further related research and clinical practice.

A total of 100 patients were enrolled in this study, focusing on the relationship between CXCR4 and GEP-NENs G3, in order to better distinguish NET G3 and NEC. We found that tumor primary location (P=0.002), and Ki-67 index (P<0.001) were significantly different between NET G3 and NEC. The expression level of CXCR4 in GEP-NENs G3 tumors and adjacent tissues was significantly different (P<0.001). In the CXCR4 low expression group, NET G3 accounted for 29.3% (24 cases) and NEC accounted for 70.7% (58 cases). In the CXCR4 high expression group, 17 cases (94.4%) were NEC and only 1 case (5.6%) was NET G3. And the high expression rate of CXCR4 in NEC was 5.7 times higher than that in NET G3 (22.7% *vs* 4%, P=0.038).

Considering that the distinction between NET G3 and NEC relies mainly on proliferation index and cell differentiation. Studies have explored diagnostic markers to distinguish NET G3 from NEC. Liverani et al. found that DLL3 is expressed in poorly differentiated NEC but not in NET G3 and is associated with poor prognosis. They concluded that DLL3 is considered to serve as a NEC diagnostic marker and potential therapeutic target (21). Bremer et al. similarly used immunohistochemistry to find that high expression of Enhancer of Zeste Homolog 2 (EZH2) in high-grade NENs was associated with poorer OS and was also significantly correlated with histological manifestations of NEC, proposing that EZH2 could be used as a biomarker to differentiate NET G3 from NEC (22). Therefore, effective markers to distinguish NET G3 from NEC are expected to be promising.

Muller et al. first explored the expression of CXCR4 in tumors in 2001, CXCR4 was highly expressed in breast cancer, but not expressed or low-expressed in normal breast tissues (23). Joseph Kim et al. also found that 89% of malignant melanoma patients with liver metastases and 97% of colorectal cancer patients with liver metastases expressed CXCR4. IHC staining of melanoma liver metastases and colorectal cancer liver metastases showed CXCR4 cytomembrane and cytoplasm staining pattern, while normal hepatocytes showed absent or weak staining (24). And several previous studies have explored CXCR4 expression in neuroendocrine tumors, but these studies generally took GEP-NENs as a whole rather than focusing on GEP-NENs G3, and the number of specimens included in these studies was also generally small (25–27). This study reveals that in GEP-NENs G3, the expression level of CXCR4 in tumors and adjacent tissues was significantly different (P<0.001) and in NEC was significantly higher than that in NET G3 (P=0.038).

Previous studies have confirmed that high expression of CXCR4 can promote tumor growth and metastasis (28, 29). In this study, the proportion of CXCR4 high expression in GEP-NENs G3 tumor diameter ≥5cm group was higher than that in tumor diameter < 5cm group, but the difference was not statistically significant (19.5% *vs* 15.1%, P = 0.572). Compared with the group without lymph node or liver metastasis, the proportion of CXCR4 high expression in the group with metastasis was slightly higher, but there was no statistical significance. R Marechal et al. found that the high expression of CXCR4 is an independent risk factor for poor prognosis of pancreatic cancer (30). In other cancer, there are also many studies confirmed that high expression of CXCR4 is associated with poor prognosis of patients (31, 32). However, in our study, we found that the prognosis of patients was not related to CXCR4 expression level, on matter in GEP-NENs G3 or NEC.

Epidemiological investigation showed that the common sites of GEP-NENs in western countries were small intestine, rectum, pancreas and stomach, while the common sites of NENs in China were pancreas, rectum and stomach (33). In this study, stomach was the most common primary site of GEP-NENs G3 (51.0%), followed by esophagus, pancreas and colorectal. However, only GEP-NENs G3 patients who received surgery in Qilu Hospital of Shandong University were included, which has selection biases and could not represent the overall epidemiological characteristics of them.

The Ki-67 index of NEC is usually significantly higher than that of NET G3 (34). Some studies have pointed out that the optimal cut-off value of Ki-67 for distinguishing pNET G3 from pNEC is 55% (35). Elettra Merola et al. found that Ki-67 index ≥55% is an independent risk factor for poor prognosis, and radical surgery is an effective treatment for resectable GEP-NENs G3 patients, especially for patients with Ki-67 index < 55% (36). The current study found that the proportion of Ki-67≥55% in NEC was significantly higher than that in NET G3 (P<0.01), and the proliferation activity of NEC was significantly higher than that of NET G3. And we also found that NEC patients had a significantly higher proportion of CEA abnormalities than NET G3 patients. All of these indicators indicate that NEC is more aggressive and malignant.

This study suggested that high CXCR4 expression was more likely to lead to a diagnosis of NEC, CXCR4 can be used in the diagnosis of high-grade neuroendocrine tumors. However, there are some limitations of this study. Firstly, we are a single-center retrospective study, and only patients who underwent surgery were included, which may result in selection bias, the sample size is small, especially the patients with NET G3, and contains specimens of many different tissues such as stomach, pancreas. Secondly, the follow-up time of this study is short, some patients were not observed to the occurrence of terminal events, the number of lost visits accounted for a high percentage, which caused a great deal of trouble for prognostic related research. What’s more, this study retrospectively collected histological specimens and performed IHC staining, however the staining quality of IHC was low and the CXCR4 staining scores had an impact. So, multi-center clinical studies with larger sample size and longer follow-up time are needed to further explore the clinicopathological characteristics, prognosis and reasonable treatment of GEP-NEN G3, and to further confirm the relationship between CXCR4 expression level and clinicopathological features and prognosis of GEP-NEN G3.

## 5 Conclusions

We found that the tumor location, and Ki-67 index were significantly different between NET G3 and NEC. CXCR4 was highly expressed only in tumor tissues, and low or no expression was found in the adjacent tissues. And the expression of CXCR4 in NEC was significantly higher than that in NET G3. However, we found there’s no significant association between CXCR4 expression level and patient prognosis.

## Data availability statement

The raw data supporting the conclusions of this article will be made available by the authors, without undue reservation.

## Ethics statement

This study has passed the review of the ethics Committee of Qilu Hospital of Shandong University (Approval number: QLCR20220462). The studies were conducted in accordance with the local legislation and institutional requirements. The participants provided their written informed consent to participate in this study.

## Author contributions

CP: Writing – review & editing, Conceptualization. YL: Writing – review & editing, Software, Writing – original draft. MS: Writing – original draft, Data curation, Investigation, Methodology. ZF: Investigation, Methodology, Formal analysis, Supervision, Writing – review & editing. XG: Investigation, Writing – review & editing, Validation. YM: Investigation, Writing – review & editing, Software. SL: Writing – review & editing, Formal analysis, Methodology. CG: Writing – review & editing, Conceptualization, Data curation. PS: Writing – review & editing, Methodology, Resources, Visualization. XW: Writing – review & editing, Methodology, Resources, Validation, Visualization. HZ: Writing – review & editing, Funding acquisition, Project administration, Resources, Supervision.
